# Implementation of a navigation system: Economic verification in a local hospital

**DOI:** 10.1371/journal.pone.0276996

**Published:** 2022-10-31

**Authors:** Ingrid Majerova, Pavel Michna, Marian Lebiedzik, Jan Nevima, Kamila Turečkova

**Affiliations:** 1 Department of Economics and Public Administration, Silesian University in Opava, School of Business Administration in Karvina, Karvina, Czech Republic; 2 AGEL a.s., Prague, Czech Republic; Instituto Tecnologico Autonomo de Mexico, MEXICO

## Abstract

Healthcare, like other industries, is increasingly using smart technologies, which also include intelligent/smart wayfinding navigation. The focus of this article, as part of the research project (its second step), was to analyze the economic effectiveness of the introduction of the wayfinding system in a local hospital in the Czech Republic. In the first phase/step of the project, possible variants of the solution were identified, verification of possible functioning, and a questionnaire survey was conducted among employees and patients regarding the waste of time in search of destination and preferences of various forms of navigation. Based on the above, it was decided to develop our own mobile application. To determine the effectiveness of this method of implementation of the new system, economic verification was used by the cost-benefit analysis method. Although the use of this method has not been required in the implementation of funded projects within the European Union since 2021, it was chosen for clarifying the pros and cons in many both investment and non-investment projects. In addition to the net present value calculation, the benefit cost ratio, profitability index, and payback period were used for evaluation. The time saving of medical staff, calculated on the basis of a questionnaire survey in the hospital (the first step of the project), was used as a benefit. The costs used were the salaries paid out in the research project, the investment, and the operating costs over the lifetime of the navigation system, which is estimated at eleven years. Using the above indicators, the implementation of the navigation system was found to be effective, despite the initial high costs. Based on these results, as part of the third step of the project, the navigation system will be implemented in the given hospital—the testing phase was taking place in the last quarter of 2021, and the full implementation is expected during the 2022.

## Introduction

For many years, hospitals have been faced with the problem of finding a destination for visitors of these facilities. This is associated not only with increased costs (in the form of wasted time), but also with a negative impact on patience experience and hospital staff workflows. The solution may be to implement intelligent smart navigation systems with low hardware and software costs. Internal wayfinding systems can be tailor-made for individual hospital facilities in the form of a mobile application that can guide patients, visitors, and hospital staff to their desired destination.

Using a mobile application has its advantages and disadvantages. The ever-expanding complexes of hospital buildings make existing information desks insufficient. Crowds form in front of them, which is why visitors often turn to the available staff and thus deprive them of valuable time. Signage is often incomprehensible, and the technical terminology used makes it difficult for visitors to understand. In many cases, it also does not provide enough information to find the desired goal. As a result, visitors find their way with a delay and frustration. This is followed by a chain reaction: medical personnel rushes under pressure to lose time, which causes the possibility of mistakes. If enough time is devoted, this causes delays for the employee and consequent financial consequences for the healthcare institution. Via a mobile application, visitors will be able to select a specific location and let themselves be navigated towards their point of interest. This eliminates their stress and the need to ask for directions and empowers them to navigate easily and independently to their destination. However, not all visitors (especially the elderly) can use mobile applications for navigation. Therefore, the traditional system should still work simultaneously, e.g. in the form of arrows and information boards. This is also supported by the results of the survey carried out in the first phase of the project among patients and visitors of the hospital, as well as among its staff, and the preference for the need for traditional navigation (orientation arrows and colored stripes) still prevails. However, the younger generation prefers more and more mobile/smart solutions [1]. Thus, the second phase of the project was started, namely, to find the optimal mobile navigation system for hospital facilities, including its verification of economic efficiency.

Similarly, [2] claimed that in the 90´s of the last century, the professional practice of wayfinding design simply involved devising sign systems; now the field is much broader and continues to expand to address technological developments. As technology advances and the literacy in information and communication technologies (ICT) of people continue to grow, modern navigation systems based on the use of different information and communication technologies tools and technologies can be expected [3].

In accordance with the above, the purpose of this article is to ascertain the economic valuation of the determined variant of the wayfinding navigation system, and so in a local hospital in the Moravian-Silesian region in the Czech Republic. This hospital is owned by the AGEL company, which is the most successful provider of healthcare in Central Europe. In the Czech Republic alone, AGEL currently operates twelve hospitals, a network of clinics, a network of pharmacies, laboratories, distribution companies, and other specialized health facilities. Since 2006, AGEL has also been operating in Slovakia. In the upcoming years, the interest of the AGEL group is also to play an important role in the fields of science, research, and innovation.

To ascertain the economic effectiveness of the proposed technical solution, the cost benefit analysis (CBA) was performed using the net present value, the benefit cost ratio, the profitability, and the payback period. The cost benefit analysis method is used as the most suitable tool for determining the effectiveness of using smart technologies in various areas of human life (healthcare, social work, transport, ICT, etc.). The calculations are given in Czech crowns (CZK), conversions to US dollars (USD) or Euro were not performed, as the purpose of the analysis is not the amount of costs or benefits, but their ratio. Therefore, it does not matter in which currency the calculations are performed; in general, a simple rounded conversion of 1 USD per 22 CZK can be performed.

Therefore, the motivation for writing this article was to show the suitability of economic verification for the introduction of a mobile navigation system, which can be used not only in the monitored hospital, but also in other medical facilities.

This paper makes two primary contributions. First, the first cost benefit analysis in the field of wayfinding in hospitals to verify its effectiveness was not applied yet, second–according to the first contribution, it creates support for the development and implementation of digital navigation systems in public facilities.

The remainder of this paper is organized as follows: materials and methods are described in the next section, including the current state of knowledge. The results of the empirical estimation are introduced in the other section and discussion is reported in the last section.

## Materials and methods

Verification of the economic effectiveness of newly introduced navigation systems requires not only thorough data collection and the use of correct methods, but also a thorough analysis of the current state of knowledge.

### Current state of knowledge

Given the aim and content of the article, the current state of knowledge can be divided into three levels: studies of wayfinding system, research of cost-benefits analysis, and issue of discounting which arises in longer-term projects within cost benefit analysis.

As for the first level, [4] defined a digital wayfinding system as a set of computing devices that are linked to a central server that generates and displays interactive wayfinding information (e.g., a map and directions to reach a desired destination as well as related parameters such as distance and estimated time). Wayfinding in healthcare will benefit patients, visitors, staff, and leaders by reducing medical errors, minimizing frustration, increasing satisfaction, and creating cost savings [5]. This also helps to get patients to their appointments on time so that scheduled appointments are not delayed, which costs the hospital additional money [6].

The ability to navigate successfully in healthcare facilities is an important goal for patients, visitors, and staff [7]. Hospital employees who travel to meetings at various locations in the hospital are often stopped in the hallways to give directions. These interruptions in movement can cause delays for employees and impact productivity [8]. Reducing or eliminating this hurdle (interruptions of staff) creates time-savings and helps hospital employees avoid tardiness, resulting in increased job satisfaction, productivity, and cost savings [8]. Patients who are unsuccessful in their wayfinding arrive late for appointments; these delays cause downtimes and disruptions in schedules and staffing [9].

The study of [10] found it to be important to have clear signage at every entry point and intersection of hallways. Signage should be immediately clear to visitors when entering the building using a navigation design, such as well-located service points or navigation symbols. Improvement in wayfinding offers several potential benefits for hospitals, including increased patient, visitor, and staff satisfaction and increased overall efficiency [6]. Improved wayfinding in an organization that increases patient satisfaction, family members, and friends leads to significant cost savings because it reduces waste and increases efficiency [11]. One common example is that it allows individuals to move efficiently through a facility without causing staff to stop their work to provide directions to lost staff, patients or visitors. This also helps patients to make their appointments on time so that scheduled appointments are not delayed, which costs the hospital additional money [6].

The effective use of technology is another key factor in best practices for finding the way. [12] proposed the use of wayfinding information systems in the form of mobile technology using specially customized software that would allow hospitals’ staff and visitors to interact with the technology for easier wayfinding. The use of technology allows patients, visitors, and staff to interact with various tools that provide an extra level of efficient navigation. Virtual reality maps and applications for mobile devices are two of the main types of technological advances for wayfinding [7]. Wayfinding strategies should foster effective communication with the broadest possible group, including people with a wide range of languages, intellectual abilities, ages, and social and cultural backgrounds [12]. Effective wayfinding should be an intuitive process that enables users to perceive and organize their environment in a way that allows navigation with minimal confusion [10].

According to [11], there are following benefits of effective wayfinding: The hospital construction boom offers healthcare architects and professionals the opportunity to fundamentally rethink hospital design and the way healthcare is delivered in an attempt to (a) reduce staff stress and fatigue, (b) increase effectiveness in care delivery, (c) improve patient safety, (d) reduce patient and family stress while improving patient outcomes, (e) improve overall quality of healthcare, and (f) improve overall hospital operating performance. Similarly [8] gives expected benefits in the form of significantly reduced time consumption for patients, visitors, and staff, informative interaction with help of location-based alerts for patients, improved patient and visitor communication and engagement, and improved patient safety and satisfaction.

Wayfinding problems in hospitals are so costly and stressful and have a particular impact on outpatients and visitors, who are often unfamiliar with the hospital and are otherwise stressed and disoriented. In a study of [13] conducted in a major regional 604-bed tertiary care hospital, the annual cost of the wayfinding system was calculated to be more than 220,000 USD per year in the main hospital. Much of this was the hidden cost of direction giving by people other than in-formation staff, which occupied more than 4,500 staff hours, the equivalent of more than two full-time positions. Several other studies have also documented the high cost of wayfinding problems in hospitals [14–17].

The implementation of a smart navigation system is important for all hospital facilities. However, the economy and efficiency of their implementation must be supported by figures, and the cost-benefit analysis is very suitable for this purpose. Cost benefit analysis is a conceptual framework applied to any systematic, quantitative appraisal of a public or private project to determine whether, or to what extent, that project is worth from a social perspective [18]. Cost benefit analysis differs from a straightforward financial appraisal in that it considers all gains (benefits) and losses (costs) to social agents. The European Commission [19] claims that the purpose of cost benefit analysis is to facilitate a more efficient allocation of resources, demonstrating the advantages for society of a particular intervention rather than possible alternatives. Cost benefit analysis is based on the analysis of all implicit and explicit costs and benefits, which quantifies the impact of investments on society [20]. Cost benefit analysis was developed as a subject, in order to be a practical guide to social decision-making [21]. The core of cost benefit analysis is an evaluation (ex ante or ex post) of the intertemporal socioeconomic benefits and costs of a project, all expressed in units of a welfare numeraire (usually money in terms of present value terms); the net effect on society is finally calculated by a quantitative performance indicator (the net present value, or the internal rate of return, or a benefit/cost ratio) [22].

The deep (bibliometric) mapping of using the cost benefit analysis in research was done by [23] in their last study. But even though this type of analysis is widely used, no output has yet been published on its application to hospital navigation systems. Some authors published their results of their research in other sectors. [24] used cost-benefit analysis in analyzing individual assistive technologies for wheelchair users, and they suggest more appropriate means of presenting the data generated by their systems that reflect real-world performance than existing systems. [25] used cost benefit analysis to determine the costs and benefits of airport road access wayfinding design and found this method to be an appropriate technique for this evaluation. The study by [26] estimated the potential net benefits of optimizing sea routes for society in the Baltic Sea and the North Sea; [27] carried out the research of cost benefit analysis in the field of large research infrastructures.

The last but not least issue of the current state of knowledge is discounting within cost benefit analysis. History of discounting dates back to the 12th century in the accounting of Italian monks, in 14th century was used by mercantilists, then by John Locke (myopic behavior) in 17th century and Pigou, Ramsey and Harrod (tyranny of discounting) in 20th century. A public investment project typically incurs costs and generates benefits at different points of time, and discounting is to express all of them in terms of their present value by assigning smaller weights to those that occur further away in the future than to those occurring more recently. In the 20^th^ century, the discussions on public sector discounting coincided with the rise of cost-benefit analysis in the 60s and 70s. In the 1990s, the choice of the social discount rate was brought up again in the context of finding a rate to discount the long-term environmental benefits and costs, such as those related to addressing climate changes and global warming [28]. Cost benefit analysis tries to consider all costs and benefits to society as a whole, so we can speak about social costs and social benefits and in this context about the social discounting rate (SDR) [29]. In other words, the social discount rate is the rate at which the whole community is willing to trade current benefits for future ones.

Applying a uniform discount rate to all government projects is problematic [30], and there is also significant disagreement among theorists as to its correct value [31]. In 2018, different countries used different SDRs: Yemen (1,8%), Jordan (7,3%), France (3,5%), Japan (5%) or UK 3,5%, Norway and Netherlands 4% for the shorter projects (under the 30 years). [32] claim, that a major reason the quality of cost benefit analysis varies widely is inconsistent use of the social discount rate (SDR).

However, discounting is a standard financial technique and the basis for intertemporal choice in economics [33]. Any evaluation of policies with future costs and benefits must specify a discount rate [34].

### Procedure

As mentioned above, this study is the second step in the research project and forms the basis for the implementation of a new smart (mobile) navigation system in the local hospitals of AGEL company. The task of the team was to find a suitable solution for the navigation system in the form of a mobile application. Therefore, an external solution was commissioned. The first technical solution was rejected due to its scope, and it was recommended to look for a system that will be simple, clear, and easy to use even for the elderly or visually impaired. Within the second solution, the requirements (displaying only the hospital premises, offline version, and map orientation for the phone parameters) were difficult to solve. In particular, the visual appearance of the map was too instructive and, given the target users, could unnecessarily complicate orientation. The third solution was rejected mainly due to the complicated administration and maintenance of the current data. Due to the assumption that the data in the application will be updated by non-IT staff, it was necessary to choose a solution and administration tool that will be simple and clear. The proposed solution was based on the developer’s know-how, and it was difficult to pass it on to other people who should take over the management after the development. On the basis of the above, a variant of the own solution was chosen.

The web application selected in this way allows, for example, visitors to find the shortest route according to the assignment. It can be used on any mobile device with a web browser. GPS sensors can be used to determine the position of movement between individual buildings and, to a limited extent, also in buildings. Because the possibilities of the GPS signal are limited in these buildings, there will be a QR code in clearly visible places to specify the visitor’s location. The client application for building navigation also allows visual (or voice) information about the direction to the destination. The point is that even users with various disabilities can use this system. Emphasis will be placed on the simplicity and intuitiveness of the control so that everyone can control the application.

To sum up the comparison with other similar navigation systems and to show that the selected version is advanced and innovative, we can state the following—our application is intuitive/user friendly, there is an offline version option, the system management is very simple and secured internally, and the application versions are as both visual and vocal.

A service application will be used to update the data in the server part of the application, which will allow system administrators to enter the data that have changed. In the case of a hospital, this may be a transfer of the relevant ambulance, either temporary with a time interval or a permanent transfer. Similarly, office hours can be updated depending on the situation. These changes take place online in real time, so individual users immediately use the current data for building navigation.

For visitors without mobile devices, information panels will be available with the option of finding the shortest route to the specified point of interest. These information panels can be in the form of information kiosks at the entrances to the premises or at the intersections of corridors. These panels and QR codes for positioning create a network of information navigation points in buildings according to which autonomous devices (such as an autonomous wheelchair) can also navigate in buildings in the future.

As part of the cost-benefit analysis, each project consists of three phases, as is the case with the project in which this paper was created. The first pre-investment phase took place in 2019, when activities related to the definition of the researched topic were carried out, surveys were conducted among patients and hospital staff, and a questionnaire survey was evaluated, based on which it was determined which type of navigation system will be suitable for implementation. The second investment phase was carried out in the years 2020 and 2021 (then also in 2026). As mentioned above, in 2020, three solutions were proposed, tested, and eventually eliminated; a decision was made about the navigation system’s own solution. In 2021, based on the results of the cost-benefit analysis, the necessary technical equipment was purchased (this will be completely replaced in 2026). The third operational phase will be implemented between 2022 and 2031, in which the navigation system will serve staff, patients, and hospital visitors. The project schedule is given in Table 1.

**Table 1:** Project schedule (in years).

Year Phase	2019	2020	2021	2022	2023	2024	2025	2026	2027	2028	2029	2030	2031
Pre-investment	x												
Investment		x	x					x					
Operational			x	x	x	x	x	x	x	x	x	x	x

### Materials used

As part of the pre-investment phase, the 785 medical and nonmedical employees of Vítkovice Hospital were addressed. The questionnaire survey focused mainly on the loss of staff time by explaining the way to other necessary examinations or visits to specialized departments. Due to the fact that administrative staff do not come into contact with patients and hospital visitors (loss of time stated as zero), their answers were not included in the analysis. 173 responses were received (22% of total employees). Of this number, 22 doctors out of 169 (13%), 136 nurses out of 423 (32%), 15 nonmedical staff out of a total of 193 (8%) answered.

The time savings were then averaged for 173 respondents according to the answers for individual professions and calculated according to the average wage in 2021 [35]. Data are shown in Table 2.

**Table 2:** Time and money costs associated with verbal navigation of patients (in CZK).

Position Item	Doctors	Nurses	Non-medical staff	All^a^
Number	22	136	15	173
Average minutes per day/person	8	16.44	7.6	-
Average hours per month/person	5.5	2.7	2.5	-
Average hourly wage/person	482	250	226	-
Cost per month/person	1,205	1,375	610	-
Costs per year/person	14,460	16,500	7,322	-
**Costs per year**	**318,120**	**2,244,000**	**110,000**	**2,672,120**

^a^Administrative staff stated that they are not asked, and their loss of time is thus zero, so they were not included in the analysis.

As can be seen from the table, the nurses report the greatest workload (calculated in minutes per day), and therefore the greatest loss of time. This is logical given their number and close contact with hospital visitors/patients. It is twice as much as in the case of doctors and nonmedical staff. In the case of recalculation for a period of one month, the situation/burden is worse for doctors, which, however, does not significantly affect the result—the costs are the highest per month anyway for nurses.

The costs for doctors and nurses per person and year are almost the same (660–750 USD), but due to the different number of employees, they vary throughout the year—while for doctors it is about 14,500 USD, for nurses it is seven times more, i.e. 100,000 USD. The lowest costs are reported by nonmedical staff—5,000 USD.

### Methods used

To calculate the efficiency of an investment through the cost benefit analysis, it is necessary first to resolve the issue of discount rate and determine its correct amount. Applying a uniform discount rate, resp. Social discount rate for all government projects is problematic. It distorts investment decisions by favoring some projects over others. Without knowing the risk composition of government projects, it would also be difficult to select the best uniform discount rate (e.g., a risk-free rate or the average market rate). However, a uniform discount rate can still be the second best if obtaining project-specific discount rates is extremely difficult [30].

However, there is significant disagreement among theorists as to the correct value of the discount rate, both due to methodological disagreements regarding which inputs are relevant to determine it (the ’prescriptive’ Ramsey equation-first approach versus direct appeals to observed interest rates) and due to disagreements about the values of the key parameters in the Ramsey equation (notably, the rate of pure time preference and the consumption elasticity of utility) [31].

According to [36], projects are discounted at rates of 3 (low) to 7 (high) percent. Unfortunately, there is no consensus over them, but instead ample variety around the rate used among countries and international credit institutions. [32] claimed that if the project is intragenerational (does not have effects beyond 50 years) and there is no crowding out of private investment, then discount all flows at 3.5%. Thus, while the European Union settled rates between 5.5 and 3.5% (the French approach with a fixed discounting rate is to apply the discounting rate at 4.5%) for the 2007–2013 period, some countries such as Colombia, Bolivia, Argentina, Uruguay, Costa Rica, or India keep 12% rates, in addition to Peru (9%) or Australia (7%). The UK approach is with declining rates following terms: 3.5% for 0 to 30 years, 3.0% for 31 to 75 years, 2.5% for 76–125 years, etc. [33]. The Czech Republic discount rate was 5.7%, Hungary 8.1%, or Slovakia 7.7% in 2007–2013 [37].

The next step is to calculate several indicators, because for the cost benefit analysis it is desirable that the efficiency of the investment is based on more indicators. The following were selected for the needs of our project: net present value, benefit cost ratio, profitability, and payback period.

The net present value (NPV) is calculated according to Eq (1) as the difference between the present value and the initial investment:

$$NPV=\sum_{t=1}^{n}PV-I$$ (1)

where

$$PV=\frac{CF}{{\left(1+DR\right)}^{t}}$$ (2)

PV is the present value of the investment, I is the investment at the beginning of the project (year ’zero’), CF is the cash flow (difference between benefits and costs) and DR is the discount rate.

Another indicator is the benefit cost ratio (BCR), which summarizes the overall relationship between the relative costs and benefits of a project and is expressed by Eq (3):

$$BCR=\frac{PV\;(benefits)}{PV\;(costs)}$$ (3)

The profitability index (IR) is a dynamic indicator suitable for evaluating the effectiveness of an investment/project, so it is also used in this case. It is calculated by the ratio of the discounted value of cash flow (NPV) and the initial investment (4):

$$IR=\frac{NPV}{I}$$ (4)

The payback period (PP) calculates how long it will take for the original investment to be repaid–the lower value of result, the better, in any way must be lower than the lifetime of investment. The following Eq (5) is used for this purpose:

$$PP=\frac{I}{\frac{\sum CF}{t}}$$ (5)

The above cost-benefit analysis indicators will be specified for the project and presented in the following section of the paper.

## Results

Based on the determination of the project schedule and after determining the discount rate of 3% and evaluation indicators in the form of net present value, benefit cost ratio, profitability index, and payback period, the economic efficiency of the implementation of the navigation system in the given hospital was verified.

According to the method of cost-benefit analysis, the "costs" group included the wages costs of the project members involved in the development of the navigation system, the purchase of smart equipment and also maintenance for the life of the system. The "benefits" group included time savings for all hospital staff that participated in the first step of project (the respondents of the questionnaire survey).

The “0” year was the year 2020, in which the most suitable variant of the navigation system was selected, followed after a questionnaire survey conducted in 2019. No benefits were reported in that year, only wages costs. In 2021, the most suitable variant was chosen (own solution), and technical equipment was purchased (information kiosks, clip frames, and tablets). The system began to be tested to be fully used from 2022. While in 2020, only costs were generated, in 2021, benefits are also reported, at a reduced value because of staff time savings only for three months of the year (proportional part since the introduction of the system). In 2022, both labor costs (within the project) and maintenance costs will be reported, as the navigation system will be fully operational. From 2023, wage costs will no longer be included, only maintenance costs (in the amount of 12 thousand CZK per year), as the staff will ensure the operation of the navigation system as part of their normal work activities. After the fifth year of operation (in 2026), the technical equipment for the navigation system (kiosks, tablets, and clipart) have to be replaced due to their end of service life. The costs will therefore increase by 131 thousand CZK.

The benefits were in the form of the savings in staff time, who have so far had to explain to patients and visitors their way to other specialized workplaces in the hospital area. Due to the vastness of the premises and the remoteness of some workplaces, staff (especially nurses) were often contacted several times, which, as already mentioned, led to their "delay from the work for which they are intended".

The development of benefits and costs, including cash flow, is shown in Table 3.

**Table 3:** Benefits and costs of the project (in thousands of CZK).

Year Item	0	1	2	3	4	5
	2020	2021	2022	2023	2024	2025
Time saving		668	2,672	2,672	2,672	2,672
**Benefits**		**668**	**2,672**	**2,672**	**2,672**	**2,672**
Wages	2,270	1,367	1,175			
Equipment		131				
Maintenance			12	12	12	12
**Costs**	**2,270**	**1,498**	**1,187**	**12**	**12**	**12**
**CF**	***2*,*270***	***830***	***1*,*485***	***2*,*660***	***2*,*660***	***2*,*660***
**Year** **Item**	**6**	**7**	**8**	**9**	**10**	**11**
	**2026**	**2027**	**2028**	**2029**	**2030**	**2031**
Time saving	2,672	2,672	2,672	2,672	2,672	2,672
**Benefits**	**2,672**	**2,672**	**2,672**	**2,672**	**2,672**	**2,672**
Wages						
Equipment	131					
Upkeep	12	12	12	12	12	12
**Costs**	**143**	**12**	**12**	**12**	**12**	**12**
***CF***	***2*,*529***	***2*,*660***	***2*,*660***	***2*,*660***	***2*,*660***	***2*,*660***

Regarding the issue of discounting, we chose its lowest rate (3%), reflecting not only the length of the project, but also the low value of the inflation rate in the Czech Republic in recent years. Based on the above results of Table 3 and the set discount rate, the values of net present value (NPV), benefit cost ratio (BCR), profitability (IR), and payback period (PP) were calculated. The results are shown in Table 4.

**Table 4:** Results of the cost benefit analysis.

Item	Result	Required value	Good/bad value
**NPV**	19,375	>0	good
**BCR**	5.27	>1	good
**IR**	9.53	>1	good
**PP**	0.96	<11	good

## Discussion

As can be seen from the above results, the values of all indicators of cost benefit analysis are very good. At first glance, the result of the net present value can be shocking. However, this is due to the almost cost-free navigation system during the last eight years of its operation. As communicated by the hospital’s managers, the cost of electricity is minimal (negligible); maintenance costs (especially payroll) will not be incurred; it will be performed by the responsible employees in the course of their work activities. Extraordinary costs may arise from theft or damage of equipment or information boards with QR codes, but here we are already at the level of speculations and as follows from the "renewal investment" in 2026 (131 thousand CZK), this is not an extremely high-cost item.

The result of the benefit cost ratio shows that the benefits exceed the costs more than 5 times, the result of the profitability index is also highly satisfactory. The value of the payback period, which should always be lower than the duration of the project, indicates less than an annual return on investment.

Although only the responses of those respondents who participated in the first phase of the project were included in the cost benefit analysis, all of these indicators have results that confirm that the implementation of a smart navigation system is the right and effective investment. It can be assumed that the time savings (benefits), given the number of employees, could be much higher and the economic return much shorter. Another limitation of this study is the listing of only quantifiable benefits and costs, to which can be added the reduction of delay stress and increased patient satisfaction.

Despite the fact that the preferences of traditional navigation elements still prevail among the hospital’s patients today, we hope that smart navigation will soon find its way to them and will be accepted and used by them more and more. However, hospitals and their management must support this; not only economic efficiency is important, but also social acceptability. In this respect, we accept the view of [16] that a wayfinding system is not just about better signage or colored lines on floors, but hospitals should provide integrated systems that include coordinated elements, such as visible and easy-to-understand signs and numbers; clear and consistent verbal directions; consistent and clear paper, mail-out, and electronic information.
